# Investigation on the expression regulation of RIPK1/RIPK3 in the retinal ganglion cells (RGCs) cultured in high glucose

**DOI:** 10.1080/21655979.2021.1944456

**Published:** 2021-07-19

**Authors:** Sheng Gao, Xi Huang, Yi Zhang, Li Bao, Xiaoyue Wang, Meixia Zhang

**Affiliations:** a Department of Ophthalmology, West China Hospital, Sichuan University, Chengdu, China; b Research Laboratory of Macular Disease, West China Hospital, Sichuan University, Chengdu, China

**Keywords:** D-glucose, RGCs, RIPK1/RIPK3, diabetic retinopathy

## Abstract

Diabetic retinopathy (DR) represents the most typical complication of type 2 diabetes mellitus and one of the most primary oculopathy causing blindness. However, the mechanism of DR remains unknown. RIPK1/RIPK3, as homologous serine/threonine kinases, are key elements in mediating necroptosis and may have functions in DR development. To clarify the relationship between DR and RIPK1/RIPK3, this study established a model of apoptosis using high-glucose induced RGCs, which were treated with 7.5, 19.5, and 35 mM D-glucose for 12, 24, and 48 h, respectively. Subsequently, the expression of RIPK1/RIPK3 was determined and the protective effect of necrostatin-1 on RGCs injury induced by high glucose was explored. The results demonstrated that the expression of RIPK1 and RIPK3 in the cells was increased markedly following 12 h treatment with 19.5 mM D-glucose. Additionally, following an addition of 100 μM necrostatin-1 in 19.5 mM D-glucose medium for RGCs treatment 12 h, the protein expression of RIPK1 and RIPK3 was decreased markedly, and the number of Nissl bodies in cells was increased substantially. The findings of the present study indicated that high glucose could induce the expression of RIPK1/RIPK3, and necrostatin-1 could effectively protect RGCs from D-glucose-induced cell necrosis.

## Graphical Abstract

**Figure:**
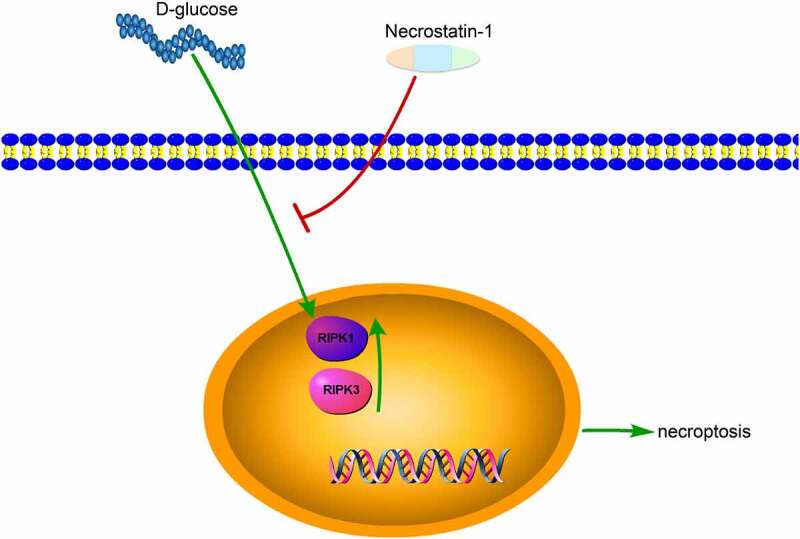

## 1 Introduction

DR is the most typical complication of type 2 diabetes mellitus, and it has become one of the most primary oculopathy causing vision loss [1]. However, the mechanism of DR remains to be fully understood. Traditionally, the pathological basis of DR is the thickening of the retinal microvascular basement membrane, resulting in loss of pericytes and impairment of blood-retinal barrier function. Increasing studies have also confirmed the pathological changes of diabetic retinal vessels, including the formation of microhemangioma, absence of capillary perfusion injury, vascular basement membrane thickening, neovascularization, and selective loss of pericytes [2–4]. However, when angiopathy occurs presenting apparent symptoms in the eyes, it represents the best time for retinopathy treatment has lost. Presently, increasing concerns have been shown on the changes of retinal neurons in early DR. RGCs are the earliest neurons differentiated from the retina, and essential in the perception, processing, and transmission of visual signals, accounting for most of the retinal nerve tissue [5,6]. Post optic nerve injury, the degree of visual acuity recovery is intimately associated with the number of survival RGCs, that is, the more survival nerves, the better recovery of visual acuity. Measures on how to save the dying RGCs and how to prevent secondary injury to RGCs have always been a hotspot and challenge for researchers. To solve this challenging scientific problem, it is necessary to track down the source to figure out the mode of RGCs necrosis and the key factors that trigger this mode of necrosis.

RIPK1 and RIPK3 are homologous serine/threonine kinases, and they are key elements in mediating necroptosis [7,8]. Besides, the activation of a series of innate immune receptors TNFR1, IFNR, and TLR, can also activate RIPK1 and RIPK3. Some studies have revealed that these stimuli can cause necroptosis in vitro [9,10]. Accumulative studies have indicated that the stimulation of TNF-a can induce necroptosis, and the activation of RIPK3 is a pivotal factor during the process [11,12]. RIPK3 can bind to RIPK1 and form a structure of ‘necrosome’, leading to the activation of downstream MLKL, and the latter serves as a key regulator in cell lysis and necrosis [13,14].

RGCs are the first cells affected in the early stages of DR and have the highest apoptosis rate. Hyperglycemia plays an important role in the development of DR disease. Hyperglycemia increases peroxides in retinal cells, promoting impaired cell viability and apoptosis, which is often accompanied by retinal ischemia and necroptosis [15]. Studies have shown that intraocular prophylactic injection of necrostatin-1 in rats protects inner retinal neurons and improves visual function [16]. In addition, RIP3 has been found to phosphorylate death-related protein (Daxx) in RGCs, causing Daxx to translocate from the nucleus and activate JNK-mediated cell death [17]. However, few reports have been made on the interaction between RIPK1/RIPK3 and high glucose.

We hypothesized that high glucose culture conditions could modulate the expression of RIPK1/RIPK3 in RGCs, resulting in RGCs damage and a reduction in niche vesicles. This study aimed to clarify the regulation of high glucose culture conditions on the expression of RIPK1/RIPK3, RGCs were subsequently isolated and cultured, cells were treated with different concentrations of D-glucose, and the changes of RIPK1/RIPK3 expression in the cells were detected.

## 2 Materials and methods

### 2.1 Primary isolation of RGCs in mice

Eighteen C57BL/6 mice were decapitated and the heads were placed in 75% alcohol for 5 min. Briefly, the eyeballs were removed gently and rinsed in D-Hank’s solution 3 times. Under an anatomical microscope, the cornea was sheared from the corneaoscleral limbus, the lens and vitreous were dissociated, and the retinal neuroepithelial tissue was bluntly separated, rinsed in D-Hank’s solution 3 times, placed into a 2 mL 0.25% trypsin solution, and incubated in a 37°C incubator for 30 min. DMEM-F12 culture medium containing 10% fetal bovine serum was added to terminate the digestion. After 1 000 rpm centrifugation for 5 min, the supernatant was discarded and NeurobasalTM Media serum-free culture system was added, which was blown with a blunt straw to prepare cell suspension. Following the cell density was adjusted to 1 × 10^5^/mL, the cells were inoculated into 24-well plates, supplemented with 1 mL of serum-free culture system containing NeurobasalTM Media to each well, and placed in a CO_2_ cell incubator maintaining a constant temperature at 37°C [18].

### 2.2 Identification of RGCs in mice by flow cytometry

The cells were cultured in 6-well plates, and when cell growth reached approximately 85%, the old culture medium was discarded. The cells were subsequently washed twice with 5 mL PBS, digested with 1 mL 0.25% trypsin until turned round, and terminated by adding 2 mL DMEM-F12 complete medium. All adherent cells were blown down, gently dispersed, transferred to a 15 mL centrifuge tube for centrifugation at 1 000 rpm for 5 min. Following that, the supernatant was carefully aspirated, it is acceptable with 50 μL medium left. Following the addition of 1 mL pre-cooled PBS, the cells were resuspended, transferred to a 1.5 mL centrifuge tube, and repeated centrifugation and precipitation. The supernatant was carefully aspirated and it is acceptable with approximately 50 μL PBS left. The bottom of the centrifuge tube was flicked gently to disperse the cells avoiding clumps. A 5 μL of Thy1.2 antibody was added to a flow cytometry tube and a 50 μL Staining Buffer to an empty tube [19]. To each tube, 50 μL cell suspension was added (approximately 10^6^ cells), mixed gently, and incubated at 4°C for 20 min. After incubation, 1 mL staining buffer was added. Following centrifugation at 1 000 rpm for 5 min, the supernatant was discarded and washed again. The cells were resuspended in 100 μL PBS, supplemented with 5 uL 488 fluorescent antibodies in the flow cytometry tube, and incubated in a refrigerator at 4°C for 20 min. After 1 000 rpm centrifugation for 5 min, the supernatant was discarded and repeated wash three times. The cells were resuspended in 100 μL PBS and detected by flow cytometry.

### 2.3 RGCs treated with different concentrations of D-glucose

When the cell density in the culture flask reached approximately 85%, the culture medium was removed. The cells were washed twice with 5 mL PBS, added 1 mL 0.25% trypsin for digestion until turned round, and then supplied with 2 mL DMEM-F12 complete medium for digestion termination. All adherent cells were blown down, gently dispersed, and transferred to three 15 mL centrifuge tubes for centrifugation at 2 000 rpm for 3 min. The cells were resuspended with the three concentrations (7.5, 19.5, and 35 mM) of D-glucose, inoculated in 6-well plates, and placed in a CO_2_ cell incubator keeping a constant temperature at 37°C for 12, 24, and 48 h [20]. Samples were collected at 12, 24, and 48 h for Western blot assays.

### 2.4 Western blot

Cell samples were lysed with lysate on ice for 30 min, centrifugated at 13,000 g for 15 min, and the supernatant was collected. The protein concentration was determined using the BCA method, diluted with sample buffer, and boiled for 5 min at 100°C. The protein was separated in 10% SDS-polyacrylamide gel via electrophoresis at 80 V for 2 h and subsequently transferred to a PVDF membrane, blocked with 5% skimmed milk for 1 h at room temperature, and incubated with primary antibodies at 4°C overnight. Following three cycles of membrane washing with TBST, 10 min each time, secondary antibodies were added and incubated at room temperature for 1 h. Following three cycles of membrane washing with TBST, 10 min each time, ECL reaction solution was added, and Bio-Rad developer was used for development. The Image Lab software was employed for quantitative analysis of the bands [18]. The antibodies used in this study were presented as follows: p-RIPK1 (1:500, Abclonal, China), RIPK1 (1:1000, Abclonal, China), p-RIPK3 (1:1000, Abclonal, China), RIPK3 (1:1000, Abclonal, China), β-Actin (1:2000, Abclonal, China) and HRP Goat Anti-Rabbit IgG (1:1000, Abclonal, China).

### 2.5 Immunofluorescence detection

The slides were placed into 24-well plates and washed three times with PBS. When the cell density reached approximately 85%, the medium was removed, washed twice with 5 mL PBS, subsequently digested with 1 mL 0.25% trypsin for digestion until turned round, and then supplied with 2 mL DMEM-F12 complete medium for digestion termination. All adherent cells were blown down, gently dispersed, and transferred to a 15 mL centrifuge tube for centrifugation at 2 000 rpm for 3 min. The cells were fixed with paraformaldehyde for 20 min and washed three times with PBS for subsequent immunofluorescence staining. The fixed cell slides were washed with distilled water and soaked in PBS for 5 min. The cells on the slides were added 0.5% TritonX-100 by drips and drilled at room temperature for 20 min. The sections were soaked with PBS for 3 times, 3 min each time. The cells were added to 10% normal goat serum and sealed at room temperature for 60 min. The blocking solution was removed with absorbent paper free of washing. The cells on each slide were added enough diluted primary antibodies (ratio: 1:200) by drips, placed into a wet box, and incubated overnight at 4°C [21]. The wet box was taken out and rewarmed at room temperature for 30 min. The slides were soaked and washed with PBS three times, 3 min each time, dried the excess liquid with absorbent paper, added diluted fluorescent secondary antibodies (ratio: 1:200), incubated in the wet box at 37°C for 60 min, and then rinsed with PBS 3 times, 3 min each time. DAPI was dripped and incubated for 5 min avoiding light. The samples were dyed and the excess DAPI was washed off with PBS for 5 min × 4 times. The Guangzhou Mingmei positive fluorescence microscopy imaging system was used to photograph.

### 2.6 Nissl staining for ganglion cell observation

The cells were fixed with paraformaldehyde for 20 min, washed with PBS three times, and changes in ganglion cell count were observed in the experimental group and the control group [22]. The cell slides were rinsed with distilled water for 3 times, 5 min each time, placed in a 60°C incubator, and stained with 1% toluidine blue for 40 min. The dye was rinsed with distilled water, and the slides were dehydrated in ethanol at 70%, 80%, 95%, and 100%, respectively, followed by xylene transparency, and finally sealed with neutral gum. The paraffin sections of gastric tissue were photographed using a Mshot MF53 microscope from Guangzhou Mingmei Optoelectronic Technology Co., Ltd.

### 2.7 Statistical analysis

Statistical analysis was performed using GraphPad Prism 8 (GraphPad Software, USA). T-tests were used for pairwise comparison and one-way analysis of variance (ANOVA) was employed for comparisons among multiple groups. The value of P less than 0.05 was considered the difference was significant [23].

## 3. Results

### 3.1 Successful isolation and identification of RGCs in mice

RGCs are the first cells affected in the early stages of DR and have the highest apoptosis rate. We hypothesized that high glucose could damage RGCs by regulating the expression of RIPK1/RIPK3. In this study, we added high glucose to RGCs and examined its regulation of RIPK1/RIPK3 expression and phosphorylation levels, and assayed Nissl bodies in RGCs.

**Figure 1.**
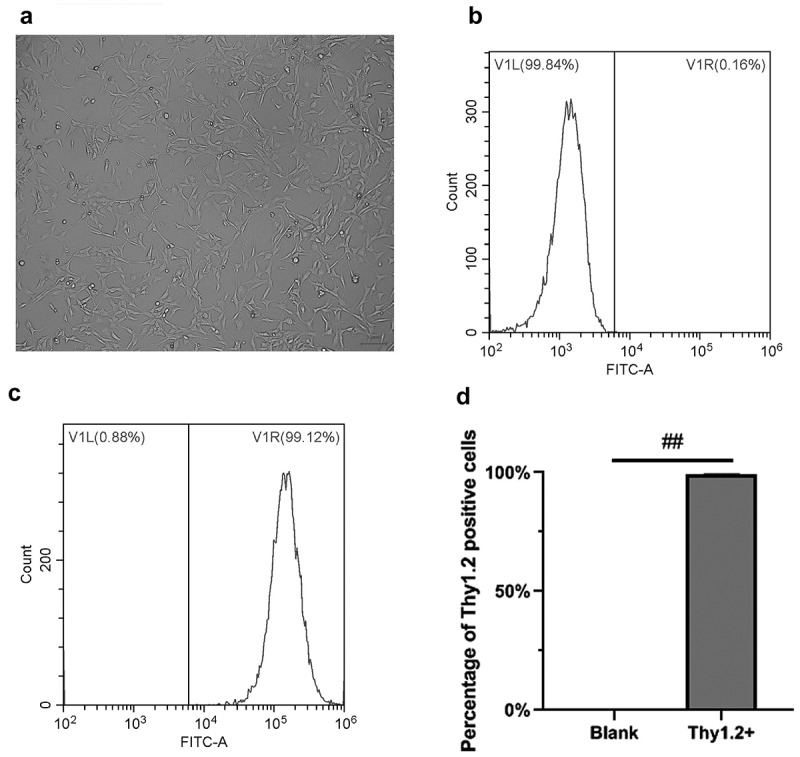
Isolation and identification of RGCs in mice. A, RGCs in mice under white light. B, Unstained cells were used as a blank internal reference to eliminate systematic errors. C, Positive rate of Thy1.2 cells by flow cytometry, the Thy1.2 antibody labeled treatment group. D, Statistical chart of the results by flow cytometry. The scale bar is 100 microns. ##, p < 0.01

Thy1.2 positive cells were detected by flow cytometry. The results indicated that unstained cells were used as a blank internal reference to eliminate systematic errors. The percentage of Thy1.2 positive cells isolated in this study was 99.18% (Figure 1), indicating that high purity RGCs in mice were isolated and could be used for subsequent studies.

### 3.2 Detection of RIPK1 and RIPK3 after D-glucose treatment

**Figure 2.**
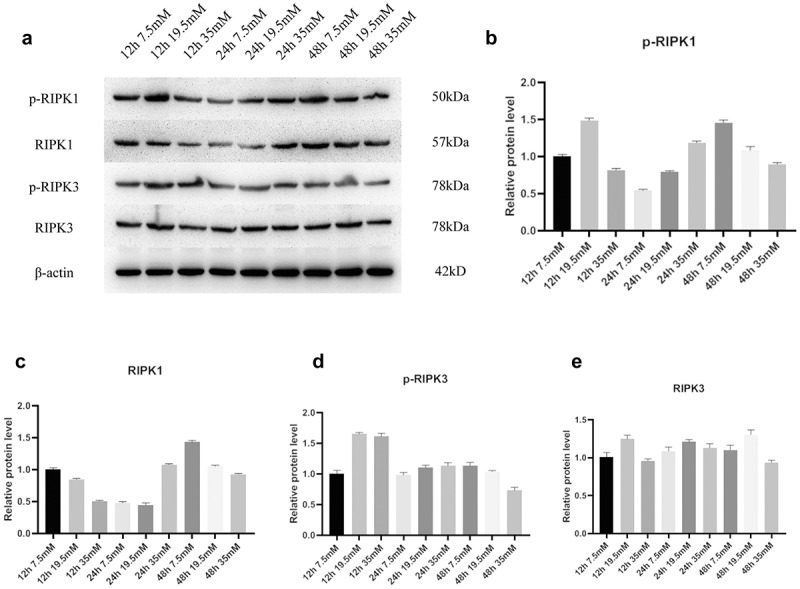
Expression of RIPK1 and RIPK3 proteins in RGCs was promoted following D-glucose treatment. A, Protein expressions of RIPK1, p-RIPK1, RIPK3, and p-RIPK3 were detected by WB after treatment of 7.5 mM, 19.5 mM, and 35 mM D-glucose for 12, 24, and 48 h. B-E, Grayscale statistics

To determine the optimal concentration and time of D-glucose on RGCs, which was applied at 7.5, 19.5, and 35 mM in treatment for 12, 24, and 48 h, respectively, and the protein expressions of RIPK1, P-RIPK1, RIPK3, and P-RIPK3 were detected. The results indicated that the expression and phosphorylation levels of RIPK1 and RIPK3 were the highest after treating 19.5 mM D-glucose for 12 h (Figure 2).

**Figure 3.**
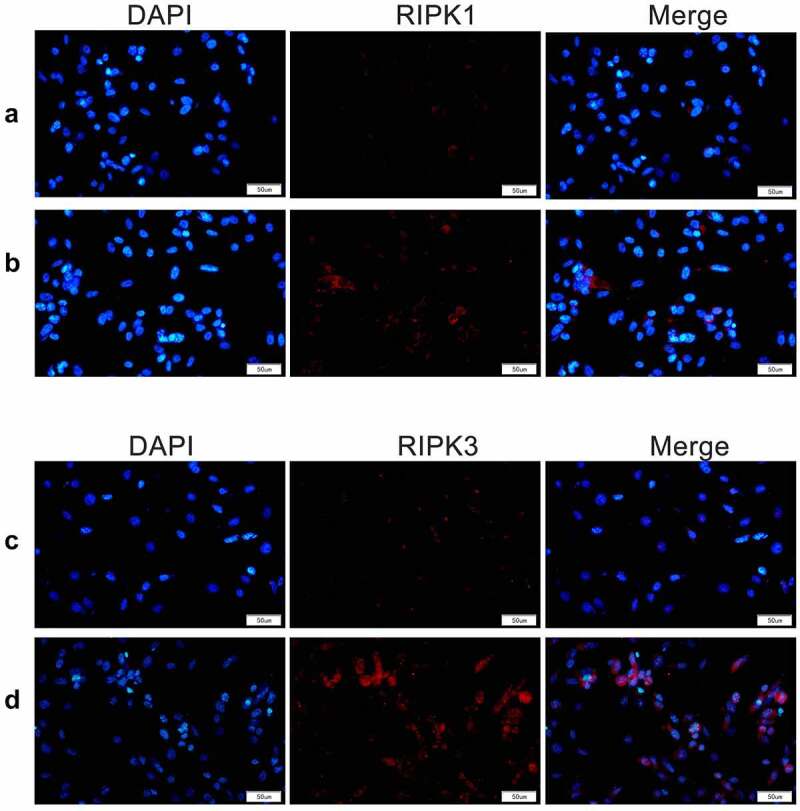
RIPK1 and RIPK3 were detected by immunofluorescence assay. Expression and distribution of RIPK1 and RIPK3 in cells were detected by immunofluorescence following treatment with 19.5 mM D-glucose at 12 h

Further verification by immunofluorescence indicated that the expression of RIPK1 and RIPK3 in the cells increased markedly following 19.5 mM D-glucose treatment at 12 h. RIPK1 and RIPK3 were distributed in both cytoplasm and nucleus (Figure 3).

### 3.3 Necrostatin-1 inhibits the expression of RIPK1 and RIPK3

**Figure 4.**
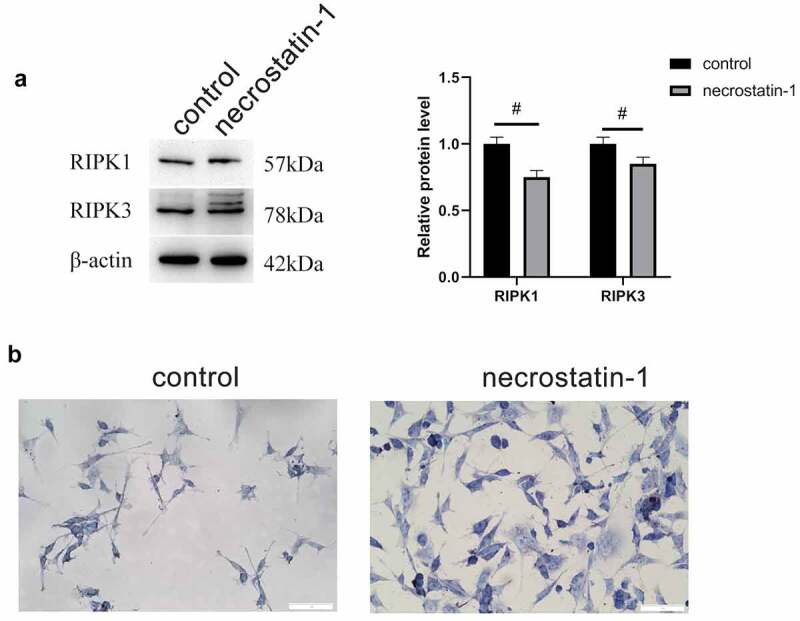
Necrostatin-1 inhibited the expression of RIPK1 and RIPK3. A, Protein expression levels of RIPK1 and RIPK3 by WB. B, Number of Nissl bodies by Nissl staining. #, p < 0.05

We subsequently added necrostatin-1 to RGCs treated with D-glucose to observe the changes in the expression of RIPK1 and RIPK3. WB assay results indicated that following the addition of 100 μM necrostatin-1 in 19.5 mM D-glucose medium, RGCs were treated for 12 h, and the protein expression of RIPK1 and RIPK3 decreased markedly (Figure 4a). Nissl staining results indicated that the addition of necrostatin-1 markedly increased the number of Nissl bodies in cells (Figure 4b), indicating that necrostatin-1 could effectively protect RGCs from D-glucose-induced cell necrosis.

## 4 Discussion

To clarify the induction effect of high glucose on RGCs and the regulation of RIPK1 and RIPK3 expression, D-glucose at 7.5, 19.5, and 35 mM was added to the isolated RGCs and cultured in vitro for 12, 24, and 48 h. By detecting the protein expressions of RIPK1, p-RIPK1, RIPK3, and p-RIPK3, the expression levels of RIPK1, RIPK3, and the phosphorylation levels were the highest after 19.5 mM D-glucose treatment at 12 h, presenting the most favorable induction effect. Further addition of necrostatin-1 to RGCs treated with D-glucose revealed that the protein expression levels of RIPK1 and RIPK3 were markedly decreased. Besides, necrostatin-1 could markedly increase the number of Nissl bodies in cells, effectively protecting RGCs from cell necrosis induced by D-glucose.

DR often results in retinal ischemia, which can lead to visual field defects or blindness. The nature of ischemia is actually due to insufficient blood supply of tissues and organs, resulting in hypoxia and ischemia of the corresponding tissues or organs. As a consequence, a series of changes occur in the intracellular environment inducing cell death. As a high metabolic organ, the retina consumes a large amount of oxygen in the human body, so the retina is very sensitive to ischemia and hypoxia [24,25]. In a recent study, Du et al. used high-glucose DMEM to establish a retinal ischemia/reperfusion (I/R) injury model in retinal ganglion cells. They focused on the regulatory mechanism of ligustrazine in this model. The results showed that low concentrations of ligustrazine significantly increased cell viability and inhibited autophagy and apoptosis of RGC-5 cells after I/R injury, indicating that low concentrations of ligustrazine had a protective effect on retinal I/R injury. In addition, the alleviating effect of ligustrazine on retinal I/R injury was mechanistically related to the activation of the PI3K/Akt/mTOR pathway. Low concentrations of ligustrazine had a significant protective effect on retinal I/R injury in RGC-5 cells through activation of the PI3K/Akt/mTOR pathway [18]. In our study, however, we focused on the construction of an in vitro model of DR using high sugar medium to investigate the mechanism of the damaging effect of high glucose on RGCs. We found that high glucose regulates the expression of RIPK1/RIPK3, leading to a decrease in Nissl bodies. In addition, we found that Necrostatin-1 was able to inhibit the high expression of RIPK1/RIPK3 induced by high glucose. We resolved the possible molecular regulatory mechanisms of DR from a novel perspective and provided new research ideas for the treatment of DR.

Once hypoxia or ischemia occurs in the retina, it will cause damage to nerve cells. Necroptosis is a way of cell necrosis by phosphorylating mixed lineage kinase domain as mediated by receptor interacting kinase, resulting in cell membrane damage and loss. Some researchers have found that necroptosis can occur in acute retinal ischemia-reperfusion injury and lead to a series of inflammatory reactions [26,27]. More studies have pointed out that RGC apoptosis can be detected as early as 7 days after retinal ischemia-reperfusion injury caused by acute intraocular hypertension [28,29]. Early in 2005, researchers found that necrostatin-1, the first inhibitor of necroptosis, became a tool protein for an actual study of necroptosis by acting on RIPK1. The amyloid-like structure of the RIPK1-RIPK3 heterodimer acts as a scaffold to recruit signaling proteins for proximity and activation. RIPK1-RIPK3 interaction alone cannot cause programmed death because it cannot transmit death information and activate downstream signals alone. Programmed necrosis is dependent on the interaction of RIPK1 and RIPK3 kinase activities, resulting in phosphorylation of the RIPK1 Ser161 site and RIPK3 Ser199 site, and only the phosphorylated RIPK3 can further perform programmed necrosis [30]. Necrostatin-1 is a potent, selective, and cell-permeable inhibitor of necroptosis. It acts by inhibiting the RIP1 in the necroptosis pathway. Necrostatin-1 inhibits overexpression and endogenous autophosphorylation of RIP1. RIP1 is the primary cellular target responsible for the anti-necrotic activity of Necrostatin-1. Necrostatin-1 effectively inhibits many types of cell-triggered Necrotropic cell death. Necrostatin-1 acts as a small molecule inhibitor of cell necrosis and inhibits the induction of cell necrosis by RIPK. Studies on the relationship of necrostatin-1 with RIPK1 and RIPK3 are well established [31]. For example, necrostatin-1 together with RIPK1 and RIPK3 plays a role in glutamate-induced toxicity in mouse embryonic fibroblasts and hippocampal neurons. After induction with glutamate, the survival of wild-type and RIP3-deficient mouse embryonic fibroblasts as well as HT22 cells was examined to probe their sensitivity to glutamate [32,33]. The present study found that the expression and phosphorylation levels of RIPK1 and RIPK3 were greatly affected in the model of RGCs induced by high glucose, and the necrotic state of RGCs could be markedly improved after RIPK1 inhibitor necrostatin-1 treatment.

RGC-5 cell line is usually used to study the neuroprotection of RGCs in vitro. Some researchers have found that calpain can upregulate apoptosis inducing factors, and lead to necroptosis of RGC-5 cell line in both models of hypoxia and glucose deprivation injury and increased hydrostatic pressure. Meanwhile, additional researchers have proved that necroptosis of the RGC-5 cell line is achieved by upregulating RIPK3 [34,35]. The researchers have discovered some specific drugs Timosaponin B and sulfur antioxidant P5. Timosaponin B is a monomer extract of the Chinese medicine Artemisia artemisiae. It can prevent the RGC-5 cell line from necroptosis by inhibiting the accumulation of tumor necrosis factor α and reactive oxygen species [36]. However, sulfur antioxidants prevent necroptosis of RGC-5 cell lines by eliminating reactive oxygen species [37]. These drugs protect cells by blocking the signaling pathway that causes necroptosis from the beginning [38]. However, few experiments have been conducted on either of the drugs in vivo [39]. Furthermore, the RGC-5 cell line is more like poorly differentiated neural progenitor cells instead of RGCs. Therefore, the in vivo experiments are more convincing than the in vitro experiments. In this study, we established a method for isolation, culture, and identification of primary RGCs. The isolated and cultured primary RGCs were adopted for more realistic in vitro experiments. It can be served as a cell material for further investigations.

Increasing studies on the discovery of necroptosis have been conducted during neuroprotection. Certainly, the pathogenesis of multiple diseases related to the central nervous system is linked to necroptosis. Compared with the central nervous system, the neurons of the retina present several similarities, mainly because the optic nerve is regarded as an extension of the central nervous system. However, as more information about the ophthalmic nerves is recognized, the role of necroptosis in ophthalmic diseases has been constantly revealed. Meanwhile, the relationship between necroptosis and apoptosis in nervous system cell death remains unclear. Luckily, our future research on these problems will assist further understanding of this approach and provide a valuable way to explore the role of necrosis in human diseases. The intervention of RIPK1 and necroptosis may be a novel method for the treatment of diseases related to neurodegeneration and cell death.

## 5. Conclusions

After the cells were treated with 19.5 mM D-glucose for 12 h, the expression of RIPK1 and RIPK3 increased substantially. High glucose can induce the expression of RIPK1/RIPK3, while necrostatin-1 can effectively protect RGCs from D-glucose-induced cell necrosis.

## Highlights

This study established a model of apoptosis using high-glucose induced RGCs.

The expression of RIPK1/RIPK3 in RGCs increased after D-glucose treatment.

Necrostatin-1 suppressed RIPK1/RIPK3 overexpression in RGCs under high glucose induction.

## References

[1] Reutrakul S, Crowley SJ, Park JC, et al. Relationship between intrinsically photosensitive ganglion cell function and circadian regulation in diabetic retinopathy. Sci Rep. 2020;10(1):1560. . 32005914 10.1038/s41598-020-58205-1 PMC6994721

[2] Sirisreetreerux S, Sujirakul T, Nimitphong H, et al. Sleep variability, 6-sulfatoxymelatonin, and diabetic retinopathy. Sleep Breath. 2020;25(2):1069–1074. . 10.1007/s11325-020-02165-3 32951070

[3] Ou K, Copland DA, Theodoropoulou S, et al. Treatment of diabetic retinopathy through neuropeptide Y-mediated enhancement of neurovascular microenvironment. J Cell Mol Med. 2020;24(7):3958–3970. . 32141716 10.1111/jcmm.15016 PMC7171318

[4] Morjaria R, Alexander I, Purbrick R, et al. Impact of Diabetic Retinopathy on Sleep, Mood, and Quality of Life. Invest Ophthalmol Vis Sci. 2019;60(6):2304–2310. . 31117122 10.1167/iovs.18-26108 PMC6532697

[5] Madrakhimov SB, Yang JY, Kim JH, et al. mTOR-dependent dysregulation of autophagy contributes to the retinal ganglion cell loss in streptozotocin-induced diabetic retinopathy. Cell Commun Signal. 2021;19(1):29. . 33637094 10.1186/s12964-020-00698-4 PMC7913405

[6] Zhou HR, Ma XF, Lin WJ, et al. Neuroprotective role of GLP-1 analog for retinal ganglion cells via PINK1/parkin-mediated mitophagy in diabetic retinopathy. Front Pharmacol. 2020;11:589114. 33679385 10.3389/fphar.2020.589114 PMC7928389

[7] Speir M, Lawlor KE. RIP-roaring inflammation: RIPK1 and RIPK3 driven NLRP3 inflammasome activation and autoinflammatory disease. Semin Cell Dev Biol. 2021;109:114–124. 32771377 10.1016/j.semcdb.2020.07.011

[8] Martens S, Hofmans S, Declercq W, et al. Inhibitors Targeting RIPK1/RIPK3: old and New Drugs. Trends Pharmacol Sci. 2020;41(3):209–224. . 32035657 10.1016/j.tips.2020.01.002

[9] Xu Y, Gao H, Hu Y, et al. High glucose-induced apoptosis and necroptosis in podocytes is regulated by UCHL1 via RIPK1/RIPK3 pathway. Exp Cell Res. 2019;382(2):111463. . 31247189 10.1016/j.yexcr.2019.06.008

[10] Najjar M, Saleh D, Zelic M, et al. RIPK1 and RIPK3 Kinases Promote Cell-Death-Independent Inflammation by Toll-like Receptor 4. Immunity. 2016;45(1):46–59. . 27396959 10.1016/j.immuni.2016.06.007 PMC4956514

[11] Akara-Amornthum P, Lomphithak T, Choksi S, et al. Key necroptotic proteins are required for Smac mimetic-mediated sensitization of cholangiocarcinoma cells to TNF-alpha and chemotherapeutic gemcitabine-induced necroptosis. PLoS One. 2020;15(1):e227454. . 10.1371/journal.pone.0227454 PMC6948742 31914150

[12] Zhang H, Ji J, Liu Q, et al. MUC1 downregulation promotes TNF-alpha-induced necroptosis in human bronchial epithelial cells via regulation of the RIPK1/RIPK3 pathway. J Cell Physiol. 2019; 234(9):15080–15088 10.1002/jcp.28148 PMC6590293 30666647

[13] Speir M, Nowell CJ, Chen AA, et al. Ptpn6 inhibits caspase-8- and Ripk3/Mlkl-dependent inflammation. Nat Immunol. 2020;21(1):54–64. . 31819256 10.1038/s41590-019-0550-7 PMC6923591

[14] Lalaoui N, Boyden SE, Oda H, et al. Mutations that prevent caspase cleavage of RIPK1 cause autoinflammatory disease. Nature. 2020;577(7788):103–108. . 31827281 10.1038/s41586-019-1828-5 PMC6930849

[15] Kaur C. Hypoxia-ischemia and retinal ganglion cell damage. Clin Ophthalmol. 2008;2(4):879–889. 19668442 10.2147/opth.s3361 PMC2699791

[16] Rosenbaum DM, Degterev A, David J, et al. Necroptosis, a novel form of caspase-independent cell death, contributes to neuronal damage in a retinal ischemia-reperfusion injury model. J Neurosci Res. 2010;88(7):1569–1576. . 20025059 10.1002/jnr.22314 PMC7713513

[17] Lee YS, Dayma Y, Park MY, et al. Daxx is a key downstream component of receptor interacting protein kinase 3 mediating retinal ischemic cell death. FEBS Lett. 2013;587(3):266–271. . 23260419 10.1016/j.febslet.2012.12.004

[18] Du HY, Wang R, Li JL, et al. Ligustrazine induces viability, suppresses apoptosis and autophagy of retinal ganglion cells with ischemia/reperfusion injury through the PI3K/Akt/mTOR signaling pathway. Bioengineered. 2021;12(1):507–515. . 33522374 10.1080/21655979.2021.1880060 PMC8806313

[19] Zhu J, Li P, Zhou YG, et al. “Altered energy metabolism during early optic nerve crush injury: implications of warburg-like aerobic glycolysis in facilitating retinal ganglion cell survival. Neurosci Bull. 2020;36(7):761–777. . 32277382 10.1007/s12264-020-00490-x PMC7340706

[20] Adamiec-Mroczek J, Zajac-Pytrus H, Misiuk-Hojlo M. Caspase-dependent apoptosis of retinal ganglion cells during the development of diabetic retinopathy. Adv Clin Exp Med. 2015;24(3):531–535. 26467145 10.17219/acem/31805

[21] Zhang Y, Liu J, Jia W, et al. AGEs/RAGE blockade downregulates Endothenin-1 (ET-1), mitigating human umbilical vein endothelial cells (HUVEC) injury in deep vein thrombosis (DVT). Bioengineered. 2021;12(1):1360–1368. . 33896376 10.1080/21655979.2021.1917980 PMC8806329

[22] McDowell CM, Luan T, Zhang Z, et al. Mutant human myocilin induces strain specific differences in ocular hypertension and optic nerve damage in mice. Exp Eye Res. 2012;100:65–72. 22575566 10.1016/j.exer.2012.04.016 PMC3612883

[23] Gahamanyi N, Song DG, Cha KH, et al. Susceptibility of campylobacter strains to selected natural products and frontline antibiotics. Antibiotics (Basel). 2020;9(11). DOI:10.3390/antibiotics9110790. PMC7697650 33182474

[24] Lee JY, Castelli V, Bonsack B, et al. Eyeballing stroke: blood flow alterations in the eye and visual impairments following transient middle cerebral artery occlusion in adult rats. Cell Transplant. 2020;29:2138898509. 10.1177/0963689720905805 PMC7444237 32098493

[25] Pan J, Zhao L. Long non-coding RNA histone deacetylase 4 antisense RNA 1 (HDAC4-AS1) inhibits HDAC4 expression in human ARPE-19 cells with hypoxic stress. Bioengineered. 2021;12(1):2228–2237. 34057022 10.1080/21655979.2021.1933821 PMC8806694

[26] Zhang YY, Li ZD, Jiang N, et al. [The effects and mechanism of baicalin in a mouse acute hypertensive glaucoma model]. Zhonghua Yan Ke Za Zhi. 2020;56(5):376–382. . 32450671 10.3760/cma.j.cn112142-20200107-00011

[27] Yu Z, Yanxia H, Limin G, et al. Melatonin alleviates pyroptosis of retinal neurons following acute intraocular hypertension. CNS Neurol Disord Drug Targets. 2020;19. DOI:10.2174/1871527319666201012125149. 33045971

[28] Gong Y, Cao X, Gong L, et al. Sulforaphane alleviates retinal ganglion cell death and inflammation by suppressing NLRP3 inflammasome activation in a rat model of retinal ischemia/reperfusion injury. Int J Immunopathol Pharmacol. 2019;33:1681099025. 10.1177/2058738419861777 PMC6610428 31266422

[29] Zhang R, Feng Y, Lu J, et al. lncRNA Ttc3-209 promotes the apoptosis of retinal ganglion cells in retinal ischemia reperfusion injury by targeting the miR-484/Wnt8a axis. Invest Ophthalmol Vis Sci. 2021;62(3):13. . 10.1167/iovs.62.3.13 PMC7960841 33687475

[30] Yang J, Zhao Y, Zhang L, et al. RIPK3/MLKL-Mediated Neuronal Necroptosis Modulates the M1/M2 Polarization of Microglia/Macrophages in the Ischemic Cortex. Cereb Cortex. 2018;28(7):2622–2635. . 29746630 10.1093/cercor/bhy089 PMC5998990

[31] Alshangiti AM, Togher KL, Hegarty SV, et al. The dietary flavonoid isoliquiritigenin is a potent cytotoxin for human neuroblastoma cells. Neuronal Signal. 2019;3(1):S20180201. . 10.1042/NS20180201 PMC7104307 32269833

[32] Liu T, Bao YH, Wang Y, et al. The role of necroptosis in neurosurgical diseases. Braz J Med Biol Res. 2015;48(4):292–298. . 25714887 10.1590/1414-431X20144310 PMC4418358

[33] Chu J, Liu CX, Song R, et al. Ferrostatin-1 protects HT-22 cells from oxidative toxicity. Neural Regen Res. 2020;15(3):528–536. . 31571665 10.4103/1673-5374.266060 PMC6921338

[34] Liao L, Shang L, Li N, et al. Mixed lineage kinase domain-like protein induces RGC-5 necroptosis following elevated hydrostatic pressure. Acta Biochim Biophys Sin (Shanghai). 2017;49(10):879–889. . 28981598 10.1093/abbs/gmx088

[35] Ding W, Shang L, Huang JF, et al. Receptor interacting protein 3-induced RGC-5 cell necroptosis following oxygen glucose deprivation. BMC Neurosci. 2015;16:49. 26238997 10.1186/s12868-015-0187-x PMC4524047

[36] Jiang SH, Shang L, Xue LX, et al. The effect and underlying mechanism of Timosaponin B-II on RGC-5 necroptosis induced by hydrogen peroxide. BMC Complement Altern Med. 2014;14:459. 25439561 10.1186/1472-6882-14-459 PMC4258277

[37] Chen P, Lai Z, Wu Y, et al. Retinal neuron is more sensitive to blue light-induced damage than glia cell due to DNA double-strand breaks. Cells. 2019;8(1): 68 10.3390/cells8010068 PMC6356720 30669263

[38] Chen HY, Ho YJ, Chou HC, et al. The role of transforming growth factor-beta in retinal ganglion cells with hyperglycemia and oxidative stress. Int J Mol Sci. 2020;21(18): 6482 10.3390/ijms21186482 PMC7554964 32899874

[39] Sano H, Namekata K, Kimura A, et al. Differential effects of N-acetylcysteine on retinal degeneration in two mouse models of normal tension glaucoma. Cell Death Dis. 2019;10(2):75. . 30692515 10.1038/s41419-019-1365-z PMC6349904

